# Supplementary material for: Comparison of Efficacy and Safety of Brigatinib in First-Line Treatments for Patients with Anaplastic Lymphoma Kinase-Positive Non-Small-Cell Lung Cancer: A Systematic Review and Indirect Treatment Comparison
Source: J Clin Med. 2022 May 24;11(11):2963. doi: 10.3390/jcm11112963 (PMC9181407; doi:10.3390/jcm11112963)
Supplement: Supplementary file 1 [file jcm-11-02963-s001.zip › jcm-1651382-supplementary.pdf]

Supplementary Documents

Table S1: Literature search strategy

	Search string	Hits
	**PUBMED**	
#1	non small cell lung cancer[Title/Abstract] OR non small cell lung carcinoma[Title/Abstract] OR non small cell lung carcinoma[Title/Abstract] OR non small cell lung carcinoma[Title/Abstract] OR non small cell lung cancer[Title/Abstract] OR non small cell lung carcinomas[Title/Abstract] OR NSCLC[Title/Abstract]	71238
#2	ALK-positive[All Fields] OR (ALK positive[All Fields] OR ALK-rearranged[All Fields] OR (ALK rearrangement[All Fields]	5165
#3	crizotinib[Title/Abstract] OR pf 02341066[Title/Abstract] OR pf 1066[Title/Abstract] OR pf 2341066[Title/Abstract] OR pf02341066[Title/Abstract] OR pf1066[Title/Abstract] OR pf2341066[Title/Abstract] OR xalkori[Title/Abstract]	2492
#4	ceritinib[Title/Abstract] OR ldk 378[Title/Abstract] OR ldk378[Title/Abstract] OR (nvp[All Fields] AND ldk 378[Title/Abstract]) OR ((nvp[All Fields] AND ldk[All Fields] AND 378[All Fields]) AND nx[Title/Abstract]) OR nvp ldk378[Title/Abstract] OR nvp ldk378 nx[Title/Abstract] OR zykadia[Title/Abstract]	517
#5	alectinib[Title/Abstract] OR af 802[Title/Abstract] OR af802[Title/Abstract] OR alecensa[Title/Abstract] OR alectinib hydrochloride[Title/Abstract] OR ch 5424802[Title/Abstract] OR ch5424802[Title/Abstract] OR rg 7853[Title/Abstract] OR rg7853[Title/Abstract] OR ro 5424802[Title/Abstract] OR ro5424802[Title/Abstract]	610
#6	brigatinib[Title/Abstract] OR ap 26113[Title/Abstract] OR ap26113[Title/Abstract] OR alunbrig[Title/Abstract]	217
#7	lorlatinib[Title/Abstract] OR pf 06463922[Title/Abstract] OR pf06463922[Title/Abstract] OR Lorbrena[Title/Abstract]	236
#8	ensartinib[Title/Abstract] OR X-396[Title/Abstract] OR X396[Title/Abstract]	35
#9	(randomized clinical trial[Publication Type]) OR (randomized[Title/Abstract])	823,166
#10	#1 and #2 and (#3 or #4 or #5 or #6 or #7 or #8)and #9	83
	**COCHRANE**	
#1	Non-Small Cell Lung Cancer OR Non-Small Cell Lung Carcinoma OR Non Small Cell Lung Carcinoma OR Non-Small-Cell Lung Carcinoma OR Non small Cell Lung Cancer OR Non-Small-Cell Lung Carcinomas OR NSCLC	14397
#2	ALK-positive OR ALK positive or ALK-rearranged OR ALK rearrangement	522
#3	crizotinib or xalkori or pf 02341066 or pf 1066 or pf 2341066 or pf02341066 or pf1066 or pf2341066	353
#4	ceritinib or zykadia or ldk 378 or ldk378 or nvp ldk 378 or nvp ldk 378 nx or nvp ldk378 or nvp ldk378 nx	72
#5	alectinib or alecensa or af 802 or af802 or alectinib hydrochloride or ch 5424802 or ch5424802 or rg 7853 or rg7853 or ro 5424802 or ro5424802	141
#6	brigatinib or alunbrig or ap 26113 or ap26113	89
#7	lorlatinib or Lorbrena or pf 06463922 or pf06463922	31

#8	ensartinib or X-396 or X396	20
#9	#1 and #2 and (#3 or #4 or #5 or #6 or #7 or #8)	234
#10	with Publication Year from 2008 to 2021.4.23	234
#11	In Trials	234
	**CNKI**	
#1	(Title, Keyword and Abstract: Non small cell lung cancer or NSCLC) AND (Full text: ALK positive or ALK rearrangement) AND (Title, Keyword and Abstract: Crizotinib or Ceritinib or Alectinib or Ensartinib or Brigatinib or Lorlatinib) AND (Title, Keyword and Abstract: Randomized clinical trial or randomized)	35
	**WanFang**	
#1	Title or Keyword: (Non small cell lung cancer or NSCLC) and Full text:(ALK positive or ALK rearrangement) and Title or Keyword: (Crizotinib or Ceritinib or Alectinib or Ensartinib or Brigatinib or Lorlatinib) and Full text: (Randomized clinical trial or randomized)	19

Table S2: PICOS criteria to assess studies for the SLR

PICOS elements	
**Population:**	• Locally advanced or metastatic (Stage IIIB-IV) NSCLC<br>• *ALK*-positive NSCLC<br>• Not previously received ALK-targeted therapy
**Intervention-Treatment:**	• ALK-TKIs (ALK tyrosine kinase inhibitors)
**Intervention-Comparison:**	• Chemotherapy<br>• ALK-TKIs (ALK tyrosine kinase inhibitors)
**Outcomes:**	• Hazard ratio of Progression free survival (PFS)<br>• Hazard ratio of Overall survival (OS)<br>• Objective response rate (ORR)<br>• Safety profile
**Study design:**	• Randomized controlled trials (Phase III)

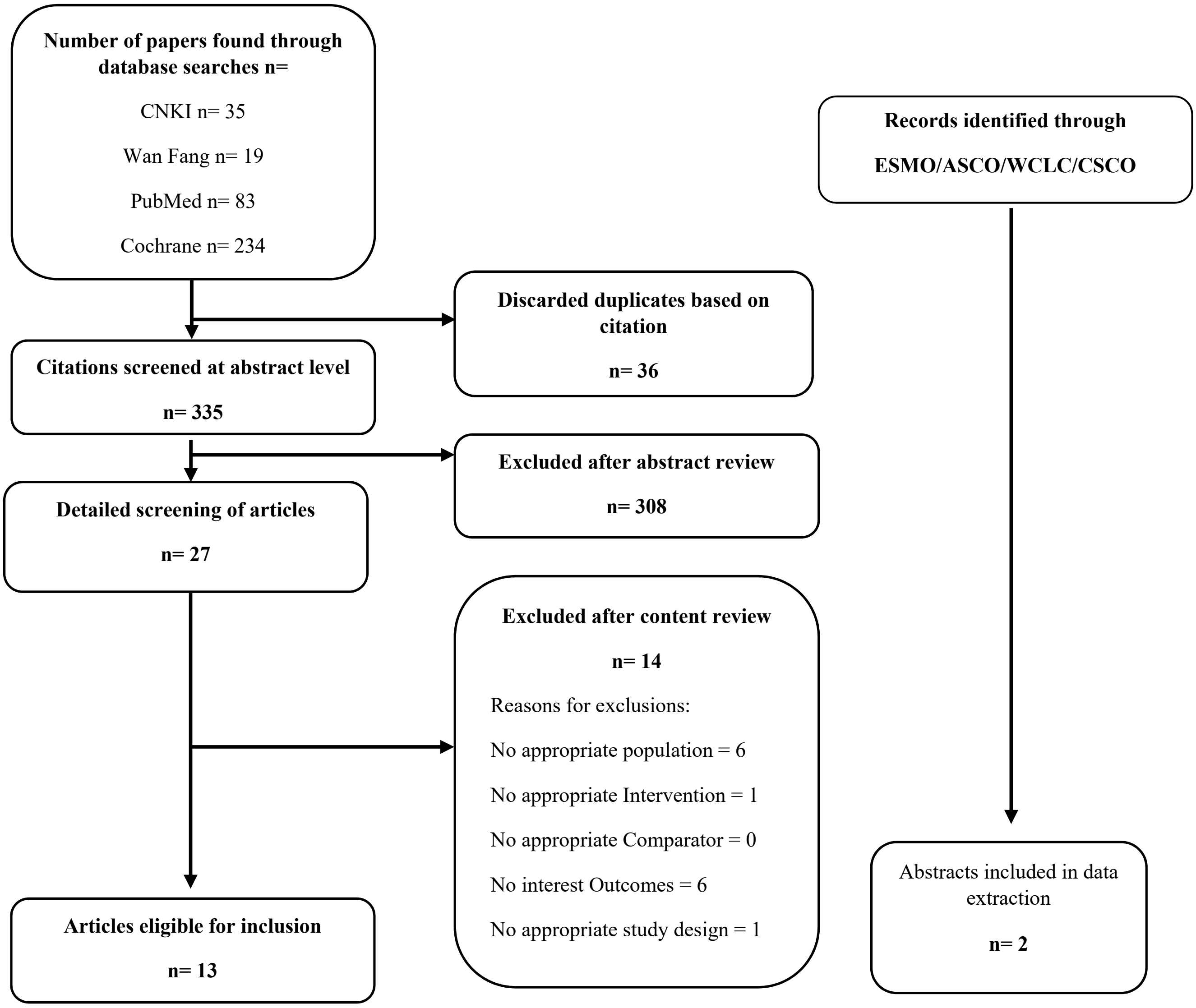

Figure S1: Flowchart of study selection

Table S3: Baseline characteristics and main outcomes of included trials

Table S3a Baseline characteristic of included trials

Author	N	Treatment	Age		Sex (%)	ECOG SCORE (%)		Disease stage (%)		
			Median	range	Male	0-1	2	ⅢB	Ⅳ	Other
D. Ross Camidge 2018[1]	137	Brigatinib	58	27–86	50%	96%	4%	6%	94%	
	138	Crizotinib	60	29–89	41%	96%	4%	9%	91%	
B. J. Solomon 2014[2]	172	Crizotinib	52	22-76	40%	94%	6%			
	171	Chemotherapy	54	19-78	37%	95%	5%			
Y L Wu 2018[3]	104	Crizotinib	48	24-67	48%	96%	4%			
	103	Chemotherapy	50	23-69	42%	96%	4%			
J. C. Soria 2017[4]	189	Ceritinib	55	22-81	46%	93%	7%	5%	95%	
	187	Chemotherapy	54	22-80	39%	94%	6%	3%	97%	
Toyoaki Hida 2017[5]	103	Alectinib	61	27–85	40%	98%	2%	3%	74%	23%
	104	Crizotinib	59.5	25–84	39%	98%	2%	3%	72%	25%
S. Peters 2017[6]	152	Alectinib	58	25–88	45%	93%	7%	3%	97%	
	151	Crizotinib	54	18–91	42%	93%	7%	4%	96%	
C. Zhou 2019[7]	125	Alectinib	51	43–59	51%	97%	3%	10%	90%	
	62	Crizotinib	49	41–59	55%	98%	2%	6%	94%	
A. T. Shaw 2020[8]	149	Lorlatinib	61	51-69	44%	98%	2%	8%	91%	1%
	147	Crizotinib	56	45-66	38%	94%	6%	5%	95%	
Leora Horn 2021[9]	143	Ensartinib	54	25-86	50%	95%	4.9%	9.1%	90.9%	
	147	Crizotinib	53	26-90	52%	95%	4.8%	6.8%	93.2%	

Author	N	Treatment	History of tobacco use (%)			Histologic features (%)		Brain metastases (%)
			Never	Current	Former	Adenocarcinoma	other	
D. Ross Camidge 2018[1]	137	Brigatinib	61%	3%	36%	92%	8%	29%
	138	Crizotinib	54%	5%	41%	99%	1%	30%
B. J. Solomon 2014[2]	172	Crizotinib	62%	6%	33%	94%	6%	26%
	171	Chemotherapy	65%	3%	32%	94%	6%	27%
Y L WU 2018[3]	104	Crizotinib	75%	6.70%	18.30%	96%	4%	20.2%
	103	Chemotherapy	70%	8.70%	21.40%	98%	2%	31.1%
J. C. Soria 2017[4]	189	Ceritinib	57%	8%	35%	95%	5%	31%
	187	Chemotherapy	65%	8%	27%	98%	2%	33%
Toyoaki Hida 2017[5]	103	Alectinib	54.0%	2.0%	44.0%	97%	3%	14.0%
	104	Crizotinib	59.0%	3.0%	38.0%	99%	1%	28.0%
S. Peters 2017[6]	152	Alectinib	61%	8%	32%	90%	10%	42%
	151	Crizotinib	65%	3%	32%	94%	6%	38%
C. Zhou 2019[7]	125	Alectinib	67.0%	3.0%	30.0%	94%	0%	35%
	62	Crizotinib	73.0%	5.0%	23.0%	97%	0%	37%
A. T. Shaw 2020[8]	149	Lorlatinib	54%	9%	37%	94%	6%	26%
	147	Crizotinib	64%	6%	29%	95%	5%	27%
Leora Horn 2021[9]	143	Ensartinib	59.40%	40.60%		NR	NR	32.90%
	147	Crizotinib	63.90%	36.10%				38.80%

Table S3b Main outcomes of included trials

Outcome	Crizotinib		Ceritinib	Alectinib			Brigatinib	Ensartinib	Lorlatinib
Publication	Full text	Full text	Full text	Full text	Full text	Full text	Full text	Full text	Full text
Trial	PROFILE 1014	PROFILE 1029	ASCEND-4	ALEX	ALESIA	J-ALEX	ALTA-1L	eXalt3	CROWN
N	343	207	376	303	187	207	275	290	296
Crossover	YES	YES	YES	NO	NO	YES	YES	NO	NO
Dosages	250 mg		750 mg	600 mg	600 mg	300 mg	180/90 mg	225 mg	100 mg
Follow-up (mo.)	45.7	22.5	19.7	48.2	16.2	68.6	40.4	23.8	18.3
PFS	10.9 (8.3-13.9)	11.1 (8.3-12.6)	16.6 (12.6-27.2)	34.8 (17.7-NE)	NE (16.7-NE)	NE (20.3-NE)	24.0 (18.4-43.2)	NR	NR
PFS, HR	0.45 (0.35-0.60)	0.402 (0.286-0.565)	0.55 (0.42-0.73)	0.43 (0.32-0.58)	0.37 (0.22-0.61)	0.37 (0.26-0.52)	0.48 (0.35-0.66)	0.5 (0.36-0.71)	0.28 (0.19-0.41)
OS	NR (45.8-NR)	28.5 (26.4-NR)	NE (29.3-NE)	NE	NE	NR	NR	NR	NR
OS, HR	0.76 (0.548-1.053)	0.897 (0.556-1.445)	0.73 (0.5-1.08)	0.67 (0.46-0.98)	0.28 (0.12-0.68)	1.03 (0.67-1.58)	0.81 (0.53-1.22); 0.50 (0.28-0.87)	0.91 (0.54-1.54)	0.72 (0.41-1.25)
ORR	74%	87.5%	72.5%	82.9%	91%	92%	74%	74%	76%

Figure S2: Risk-of-bias summary

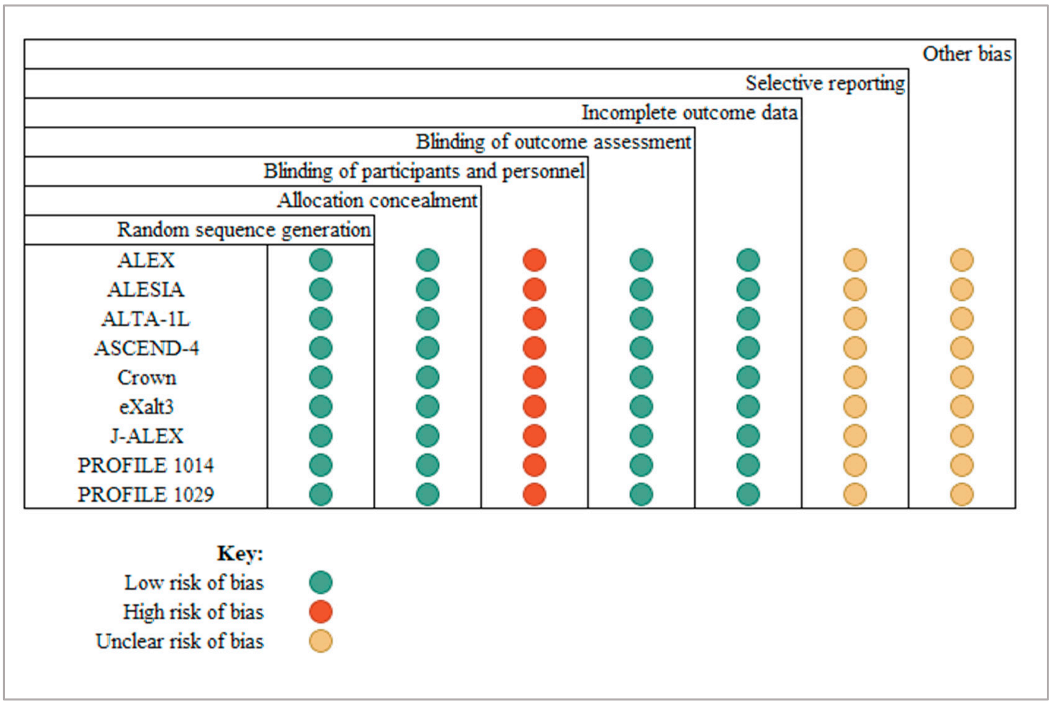

Figure S3: Brigatinib vs Alectinib (ALEX) in PFS in patients with baseline brain metastases

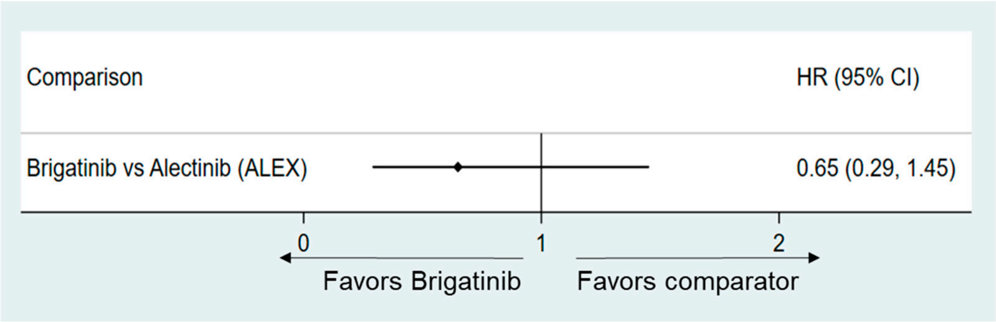

REFERENCES

1. Camidge, D.R.; Kim, H.R.; Ahn, M.-J.; Yang, J.C.-H.; Han, J.-Y.; Lee, J.-S.; Hochmair, M.J.; Li, J.Y.-C.; Chang, G.-C.; Lee, K.H.; et al. Brigatinib versus Crizotinib in ALK-Positive Non-Small-Cell Lung Cancer. *N. Engl. J. Med.* **2018**, *379*, 2027–2039. https://doi.org/10.1056/NEJMoa1810171.
2. Solomon, B.J.; Mok, T.; Kim, Do.; Wu, Yi.; Nakagawa, K.; Mekhail, T.; Felip, E.; Cappuzzo, F.; Paolini, J.; Usari, T.; et al. First-line crizotinib versus chemotherapy in ALK-positive lung cancer. *N. Engl. J. Med.* **2014**, *371*, 2167–2177. https://doi.org/10.1056/NEJMoa1408440.
3. Wu, Y.-L.; Lu, S.; Lu, Y.; Zhou, J.; Shi, Y.-K.; Sriuranpong, V.; Ho, J.C.; Ong, C.K.; Tsai, C.-M.; Chung, C.-H.; et al. Results of PROFILE 1029, a Phase III Comparison of First-Line Crizotinib versus Chemotherapy in East Asian Patients with ALK-Positive Advanced Non-Small Cell Lung Cancer. *J. Thorac. Oncol.* **2018**, *13*, 1539–1548. https://doi.org/10.1016/j.jtho.2018.06.012.
4. Soria, J.C.; Tan, D.S.W.; Chiari, R.; Wu, Yi.; Paz-Ares, L.; Wolf, J.; Geater, S.L.; Orlov, S.; Cortinovis, D.; Yu, C.; et al. First-line ceritinib versus platinum-based chemotherapy in advanced ALK-rearranged non-small-cell lung cancer (ASCEND-4): A randomised, open-label, phase 3 study. *Lancet* **2017**, *389*, 917–929. https://doi.org/10.1016/S0140-6736(17)30123-X.
5. Hida, T.; Nokihara, H.; Kondo, M.; Kim, Y.H.; Azuma, K.; Seto, T.; Takiguchi, Y.; Nishio, M.; Yoshioka, H.; Imamura, F.; et al. Alectinib versus crizotinib in patients with ALK-positive non-small-cell lung cancer (J-ALEX): An open-label, randomised phase 3 trial. *Lancet* **2017**, *390*, 29–39. https://doi.org/10.1016/s0140-6736(17)30565-2.
6. Peters S, Camidge DR, Shaw AT, et al. Alectinib versus Crizotinib in Untreated ALK-Positive Non-Small-Cell Lung Cancer. *N Engl J Med*. **2017**, 9, 829-838. doi:10.1056/NEJMoa1704795
7. Zhou, C.; Kim, S.-W.; Reungwetwattana, T.; Zhou, J.; Zhang, Y.; He, J.; Yang, J.-J.; Cheng, Y.; Lee, S.-H.; Bu, L.; et al. Alectinib versus crizotinib in untreated Asian patients with anaplastic lymphoma kinase-positive non-small-cell lung cancer (ALESIA): A randomised phase 3 study. *Lancet Respir. Med.* **2019**, *7*, 437–446. https://doi.org/10.1016/s2213-2600(19)30053-0.
8. Shaw, A.T.; Bauer, T.M.; de Marinis, F.; Felip, E.; Goto, Y.; Liu, G.; Mazieres, J.; Kim, D.-W.; Mok, T.; Polli, A.; et al. First-Line Lorlatinib or Crizotinib in Advanced ALK-Positive Lung Cancer. *N. Engl. J. Med.* **2020**, *383*, 2018–2029. https://doi.org/10.1056/NEJMoa2027187.
9. Horn L, Wang Z, Wu G, et al. Ensartinib vs Crizotinib for Patients With Anaplastic Lymphoma Kinase-Positive Non-Small Cell Lung Cancer: A Randomized Clinical Trial. *JAMA Oncol.* **2021**, 7, 1617-1625. doi:10.1001/jamaoncol.2021.3523
